# 

**Published:** 1877-05

**Authors:** 


					﻿Vol. 24.
MAY, 1877.
No. 5.
TWENTY-FOURTH YEAR.
HALL’S
J oumal of Health.
PUBLISHED AT 137 EIGHTH STREET, NEW YORK,
Four Doors East of 750 Broadway.
E. H. GIBBS, M.D., Editor.
AGENTS FOB THE TBADB:
AMERICAN NEWS COMPANY, 121 NASSAU STREET.
NEW YORK NEWS COMPANY, 18 BEEKMAN STREET.
Terms, $1.50 a Year.
SINGLE NUMBER, 15 CENTS.
Kknt & Co.. Printers, 11 Frankfort St, New York.
				

